# Loss of heterozygosity on chromosomes 11 and 17 are markers of recurrence in TCC of the bladder

**DOI:** 10.1054/bjoc.2001.2159

**Published:** 2001-12

**Authors:** J Edwards, P Duncan, J J Going, K M Grigor, A D Watters, J M S Bartlett

**Affiliations:** University Department of Surgery, University Department of Pathology, Glasgow Royal Infirmary, Glasgow, G31 2ER; University Department of Pathology, Edinburgh Royal Infirmary, Edinburgh, EH8 9AG, UK

**Keywords:** LOH, TCC of the urinary bladder, chromosome 11 and 17

## Abstract

Approximately 2/3 of patients diagnosed with superficial transitional cell carcinoma of the urinary bladder (TCC) will recur within 2 years. Loss of chromosome 9 and loss of heterozygosity (LOH) at 9q34 in index TCCs identify a subset of patients at high risk of recurrence. This study explores genetic alterations on chromosomes 4, 8, 11 and 17 as predictors of recurrence. A total of 109 carcinomas were investigated at 26 loci. DNA was extracted from microdissected archival normal/tumour tissue and was analysed for loss of heterozygosity (LOH). Fluorescent PCR was performed and genotyping carried out on a Perkin Elmer ABI377 sequencer. LOH of D11S490 or D17S928 was significantly more frequent in index carcinomas of patients who experienced recurrence compared to those with no recurrence (*P* = 0.004 and 0.019 respectively). These results suggest that loss of these regions is associated with recurrence of TCC. Further investigation is required to identify genes in these regions, which might be responsible for driving recurrence in TCC of the urinary bladder. © 2001 Cancer Research Campaign http://www.bjcancer.com


British Journal of Cancer (2001) 85(12), 1894–1899
© 2001 Cancer Research Campaign
doi: 10.1054/ bjoc.2001.2159, available online at http://www.idealibrary.com on IDEAL

### Loss of heterozygosity on chromosomes 11 and 17 are markers of recurrence in TCC of the bladder

**J Edwards[1], P Duncan[1], JJ Going[2], KM Grigor[3], AD Watters[1] and JMS Bartlett[1]**

[1]University Department of Surgery and [2]University Department of Pathology, Glasgow Royal Infirmary, Glasgow, G31 2ER; and [3]University Department of Pathology, Edinburgh Royal Infirmary, Edinburgh, EH8 9AG, UK

**Summary** Approximately 2/3 of patients diagnosed with superficial transitional cell carcinoma of the urinary bladder (TCC) will recur within 2 years. Loss of chromosome 9 and loss of heterozygosity (LOH) at 9q34 in index TCCs identify a subset of patients at high risk of recurrence. This study explores genetic alterations on chromosomes 4, 8, 11 and 17 as predictors of recurrence. A total of 109 carcinomas were investigated at 26 loci. DNA was extracted from microdissected archival normal/tumour tissue and was analysed for loss of heterozygosity (LOH). Fluorescent PCR was performed and genotyping carried out on a Perkin Elmer ABI377 sequencer. LOH of D11S490 or D17S928 was significantly more frequent in index carcinomas of patients who experienced recurrence compared to those with no recurrence ($P = 0.004$ and 0.019 respectively). These results suggest that loss of these regions is associated with recurrence of TCC. Further investigation is required to identify genes in these regions, which might be responsible for driving recurrence in TCC of the urinary bladder. © 2001 Cancer Research Campaign http://www.bjcancer.com

**Keywords**: LOH; TCC of the urinary bladder; chromosome 11 and 17

Most (80%) transitional cell carcinomas (TCC) of the urinary bladder are low stage (pTa or pT1) at initial presentation (Van der Meiden, 1999) and may be surgically removed leaving no detectable disease. However, 50–70% of these patients present with a recurrence within 2 years and 20% of the recurrences progress to detrusor muscle invasion. TCCs that invade the muscle cannot be eradicated by endoscopic surgery alone; and 50% of patients with this disease develop metastases and die within 5 years (Adshead et al, 1999).

Currently only a few genetic aberrations are associated with development and progression of TCC of the urinary bladder, e.g. loss of chromosome 9, p53 mutations and gain of *c-myc* (Bartlett et al, 1998; Tsutsumi et al, 1997; Sauter et al, 1995). Such genetic changes may determine the path a TCC will follow (Bartlett et al, 1998; Watters et al, 2000). Several studies demonstrate genetic differences between pTa and pT1 carcinomas (Richter et al, 1997; Simon et al, 1998). These studies suggest that a higher level of genetic abnormalities in pT1 carcinomas increase their likelihood of progression. It is, however, difficult for the pathologist to accurately stage pTa/pT1 carcinomas. Therefore, even if this distinction was proven, identification of the carcinoma suppressor genes that drive TCC recurrence and/or progression would not only increase our understanding of TCC disease development and progression but would also enable us to predict the path an index TCC might take.

*Received 4 July 2001*
*Revised 30 August 2001*
*Accepted 19 September 2001*

*Correspondence to:* JMS Bartlett, University Department of Surgery, Level II, Queen Elizabeth Buildings, Glasgow Royal Infirmary, Glasgow, G31 2ER, UK

Loss of heterozygosity (LOH), at the region of a known tumour suppressor gene, is considered indicative of a mutation in the remaining copy of the gene and loss of the tumour suppressing function of the gene product. LOH analysis of sequential carcinomas from TCC patients would, therefore enable tracking of tumour suppressor gene inactivation in relation to recurrence (Bartlett et al, 1998; Wagner et al, 1999; Watters et al, 2000).

Deletions involving all or part of chromosome 9 are the most frequently reported genetic event in TCC of the urinary bladder (Bartlett et al, 1998; Knowles, 1998, 1999; Czerniak et al, 1999). We have recently demonstrated that deletion of *TSC1* on chromosome 9 predicts recurrence in a subset of TCC patients (Edwards et al, 2000). However, in order to identify further recurrence-related genes, other areas of deletion associated with bladder cancer should be investigated. The current study has selected areas of deletion previously associated with TCC of the bladder. LOH at 4p16.3 has been reported in over 1/3 of bladder cancer cell lines (Bell et al, 1996) and deletions in 8p are associated with high-grade carcinomas and distant metastasis of urinary bladder cancers (Ohgaki et al, 1999). LOH on both 11p and 11 q have frequently been reported in TCC of the bladder (Fearon et al, 1985; Shaw and Knowles, 1995; Voorter et al, 1996; Monaco et al, 1997). Loss of 17p13.1, the *TP53* locus, is associated with high tumour grade (Tsutsumi et al, 1997; Esrig et al, 1993; Chaturvedi et al, 1997) and invasive growth in bladder cancer (Tsutsumi et al, 1997). Loss of 17q22 and 17q24-25 have also been reported (Chaturvedi et al, 1997).

This study investigated LOH in 109 TCCs of the urinary bladder from 46 patients with known follow-up. The patients fell into two groups: those with carcinomas that did not recur and those with carcinomas that recurred and/or progressed. LOH was investigated using a panel of microsatellite markers that spanned chromosomes 4, 8, 11 and 17.

### MATERIALS AND METHODS

### Patients and samples

Forty-six patients were selected for this retrospective study. Ethical approval was gained from the local ethics board. Patients either had non-recurrent carcinomas (NR; *n* = 17) and were disease free with follow-up for a minimum of 3 years or had recurrent carcinomas (REC; *n* = 29). These patients had several episodes of recurrent TCC after the index carcinoma.

Archival (formalin fixed, paraffin embedded) TCCs were regraded and staged by KMG using UICC criteria (UICC, 1978). Tissue sections dewaxed in xylene (2 × 10 min), and rehydrated through 99% alcohol (2 × 2 min) and 95% alcohol (2 min) were stained in 0.05% Toludine blue for 30 s. Areas of tumour were microdissected from 5 µm sections. DNA was extracted by incubating for 4–7 days at 37˚C in 25 µl of proteinase K in digestion buffer (0.5 mg/ml proteinase K, 0.5% Tween 20 in 50 mM Tris-EDTA pH 8.5) (Going and Lamb, 1996). The proteinase K was inactivated by heating at 95˚C for 10 min. Normal tissue (e.g. detrusor muscle) was also dissected from the same section. LOH analysis was conducted using 1 µl aliquots of DNA without further purification.

### PCR

Twenty-six microsatellites spanning four chromosomes were investigated (Table 1). Primers were from MWG-Biotech UK Ltd or Perkin-Elmer Applied Biosystems, with one primer from each pair fluorescently labelled at the 5′ end all primer sequences are available from the authors on request.

Target sequences were amplified by PCR in 10 µl reactions (1× reaction buffer containing 10 pmol of each primer, 3 mM $MgCl_2$, 200 µM each of dATP, dCTP, dGTP, dTTP and 0.5 units hot start Taq polymerase (Qiagen Ltd, Sussex, UK)). The reaction was started after 15 min denaturation of DNA at 95˚C. DNA amplification was performed in a MJ Research PTC – 225 Peltier Thermal Cycler (Genetic Research Instrumentation, Essex, UK) as follows: 45 cycles of denaturation at 94˚C for 30 s, annealing at 47, 49, 50, 51 or 53˚C (Table 1) for 30 s and extension at 72˚C for 1 min, followed by a final extension for 15 min at 72˚C.

### PCR analysis

Genotyping was performed on an automated laser activated fluorescent DNA sequencer (Perkin Elmer ABI 377 sequencer) by DNASHEF Technologies, Edinburgh. Fluorescent gel data were collected automatically during electrophoresis and analysed using Genescan™ software, peak size, height and area were measured. Allele loss was assessed as described by Niederacher et al, (1997), i.e. the allele ratio of carcinoma DNA peak height was divided by the allele ratio of paired normal DNA peak height. A ratio below 0.65 or above 1.55 was considered indicative of loss. Repeat analysis was performed on random samples to confirm reproducibility.

**Table 1** Outlines the microsatellites studied, their chromosomal region and the annealing temperature of their PCRs

| Microsatellite markers | Region | Temperature (˚C) |
|---|---|---|
| D4S174 | 4p11–15 | 50 |
| D4S127 | 4p16.3 | 53 |
| D8S254 | 8p22 | 50 |
| D8S133 | 8p22 | 50 |
| D10S215 | 10q23.1 | 47 |
| D11S922 | 11p15.5 | 53 |
| D11S569 | 11p15.1 | 50 |
| FGF3 | 11q13 | 53 |
| D11S490 | 11q23.3 | 50 |
| D17S578 | 17p13.3 | 49 |
| D17S849 | 17p13.3 | 50 |
| D17S960 | 17p13.1 | 49 |
| TP53 | 17p13.1 | 50 |
| D17S786 | 17p13.1 | 49 |
| D17S1852 | 17p13.1 | 50 |
| D17S799 | 17p12 | 50 |
| D17S921 | 17p12 | 50 |
| D17S1857 | 17p12 | 50 |
| D17S798 | 17q11–12 | 50 |
| D17S932 | 17q21 | 49 |
| D17S579 | 17q21 | 51 |
| D17S943 | 17q21 | 49 |
| D17S944 | 17q21 | 51 |
| D17S807 | 17q21 | 49 |
| D17S949 | 17q21 | 50 |
| D17S784 | 17q24–25 | 51 |
| D17S928 | 17q24–25 | 51 |

### Statistics

Differences between patient groups were analysed using Fisher's exact test. Disease free survival was evaluated using the Kaplan-Meier method and curves were compared with the log rank test.

### RESULTS

There were no significant differences between patient age or sex or index carcinoma stage or grade when the NR and REC groups were compared. The stage and grade of all carcinomas used were known (Table 2). The success rate of LOH analysis from archival carcinoma material was greater than 95%.

### Index carcinomas

The frequency of LOH at all informative markers spanning chromosomes 4, 8, 11 and 17 were compared in index NR and index REC

**Table 2** Shows the stage and grade of carcinomas, NR denotes non recurrers, index (REC) denotes the index carcinomas of patients with subsequent recurrences and recurrent (REC) denotes the recurrent carcinomas

| | Stage | | | | Grade | | |
|---|---|---|---|---|---|---|---|
| | Ta | T1 | T2 | Other | 1 | 2 | 3 |
| NR | 13/17 | 4/17 | | | 6/17 | 11/17 | |
| Index (REC) | 17/29 | 9/29 | | 3/29 | 11/29 | 10/29 | 5/29 |
| Recurrent (REC) | 37/63 | 11/63 | 15/63 | | 21/63 | 18/63 | 24/63 |

© 2001 Cancer Research Campaign

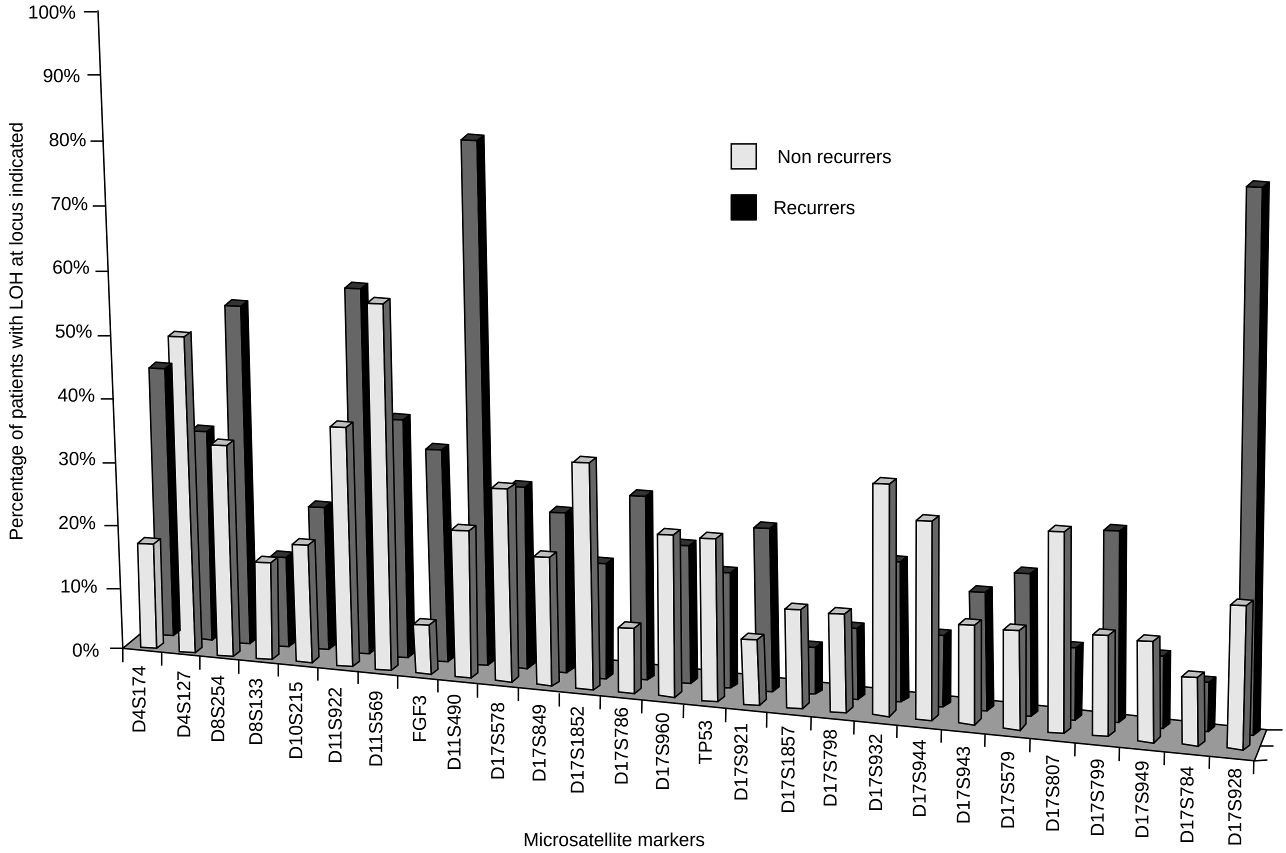

**Figure 1** LOH frequency at each loci for the index carcinomas of the NR and REC groups

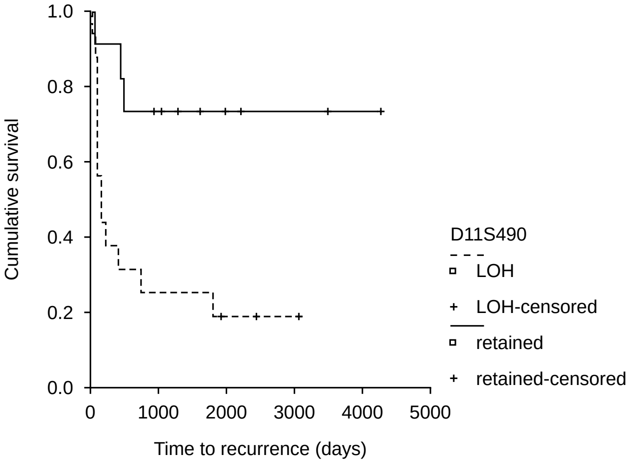

**Figure 2** Disease free survival of 28 patients who retained or had LOH at D11S490 (*P* = 0.0079)

**Table 3** Shows the frequency of LOH at all informative markers spanning chromosome 4.8, 11 and 17 in two patient groups. NR = non-recurrers and REC = recurrers

| | NR | REC |
|---|---|---|
| Chromosome 4 | 29% (5/17) | 21% (6/28) |
| Chromosome 8 | 12% (2/17) | 3% (1/28) |
| Chromosome 11 | 0% (0/17) | 18% (5/28) |
| Chromosome 17 | 0% (0/17) | 0% (0/28) |

carcinomas; no significant differences were identified (Table 3). No significant differences were identified when loss of chromosomal regions were compared for index NR and index REC carcinomas.

The frequency of loss of D11S490 was significantly higher in patients who recurred compared to patients who did not recur (NR, 25%, 3/12 versus REC, 81%, 13/16, *P* = 0.004) (Figure 1). This held true when pTa carcinomas alone were considered (NR, 20% 2/10, versus REC 89%, 8/9, *P* = 0.001). Time to recurrence was significantly longer in patients who retained D11S490 than in those who lost it (Figure 2, *P* = 0.0079); 16 patients out of 28 informative cases had LOH at D11S490.

The frequency of loss of D17S928 was significantly higher in the index carcinomas from the REC group compared to NR carcinomas (NR, 21%, 3/14 versus REC, 80%, 16/20, *P* = 0.002) (Figure 1). This held true when the pTa carcinomas alone were considered (NR, 15%, 2/13 versus REC, 83%, 10/12, *P* = 0.002). Time to recurrence was significantly longer in patients who retained D17S928 than in those who lost it (Figure 3, *P* = 0.04); 19 patients out of 34 informative cases had LOH at D17S928.

LOH data from D11S490 and D17S928 identified 23 out of the 29 patients as likely to recur compared to 13 for D11S490 and 16 for D17S928. All patients were included and none were lost due to non informative markers. Time to recurrence was significantly shorter in patients who had LOH at either D11S490 and D17S 928 than in those who had retention at either or both (*P* = 0.03).

 *© 2001 Cancer Research Campaign*

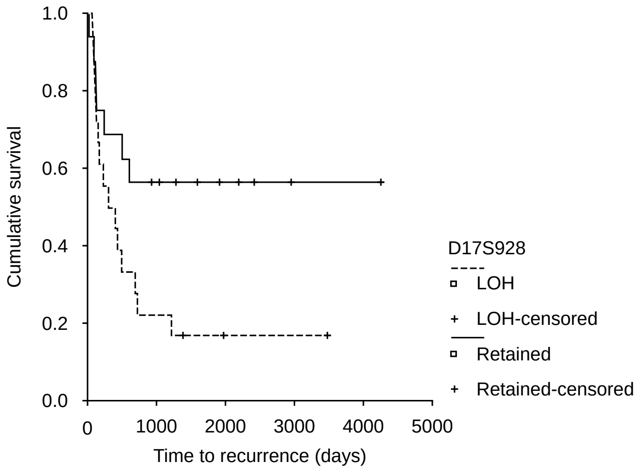

**Figure 3** Disease free survival of 34 patients who retained or had LOH at D17S928 ($P = 0.04$)

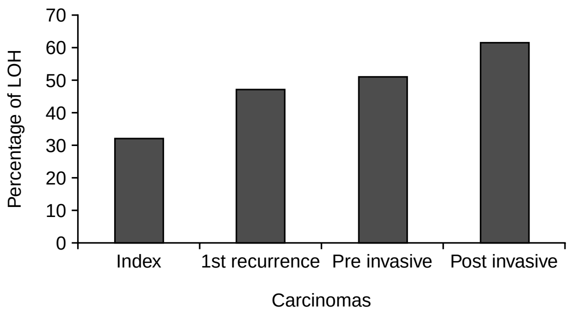

**Figure 4** Overall percentage of LOH noted in index, first recurrence, immediately pre-invasive and muscle invasive carcinomas of patients who presented with a low grade carcinoma which recurred and progressed to a muscle invasive carcinoma (RP)

### The trend in total LOH frequency as a carcinoma recurs and progresses

The trend of LOH in recurrent carcinomas was investigated, the REC group being sub-divided into carcinomas that recurred (RNP) and carcinomas that recurred and progressed to higher stage disease (RP). The frequency of LOH in each group was calculated by dividing the total number of informative cases by the total number of LOHs in each group and multiplying by 100, therefore the frequency was expressed as a percentage. In the RNP group there was no significant difference in the frequency of LOH observed in index and recurrent carcinomas (index tumour 40% (116/316), first recurrence 47% (146/310) and last recurrence 48% (117/241). In the RP patient group the frequency of LOH increased with progression: 32% (66/205) of index carcinomas, 47% (88/ 187) of first recurrent carcinomas, 51% (99/192) of immediately pre-invasive carcinomas and 61% (90/148) of post-invasive carcinomas. As the index carcinoma progressed to an invasive carcinoma a significant increase in the frequency of LOH was noted ($P > 0.0001$) (Figure 4).

### The significance of LOH levels in carcinomas grouped by grade and stage

When carcinomas were grouped by stage and the frequency of LOH compared for individual loci or chromosomal regions the only significant difference was at 8p22, pTa carcinomas had a significantly lower frequency of LOH than pT1 carcinomas (19/39, 48% versus 10/11, 91% $P = 0.024$).

When the frequency of LOH was compared for carcinomas grouped by grade, loss of all informative markers spanning the *TOP II* region on chromosome 17 was found to increase with grade. (Grade 1, 2/33, 6% versus grade 2, 9/25, 36%, $P = 0.01$; and grade 1, 2/33, 6% versus grade 2 and 3, 15/49, 25%, $P = 0.01$).

### DISCUSSION

Current molecular and pathological parameters fail to predict which early TCCs will recur and/or progress. This study assessed genetic changes on chromosome 4, 8, 11 and 17 in two patient groups in an attempt to identify genetic changes in index carcinomas that would predict the path a carcinoma might follow. The index carcinomas investigated were either pTa or pT1. The results were analysed with pTa and pT1 index carcinomas combined and separated as genetic differences between pTa and pT1 carcinomas have now been identified (Richter et al, 1997; Simon et al, 1998). Interestingly only one marker had significantly different frequency of LOH when the carcinomas were grouped by stage (8p22 between pTa and pT1) or grade (17p21 between grade 1 and grade 2). These results suggest that the frequency of LOH at the microsatellite loci used in this study is independent of stage or grade.

Frequent deletions on both arms of chromosome 11 in TCC of the urinary bladder indicate the probable location of relevant tumour suppressor gene/s (Fearon et al, 1985; Voorter et al, 1996). Distinct regions of deletion exist on 11p (11p13 and 11p15) and 11q (11q13–q23.3), and LOH in these regions is associated with high grade carcinomas (Shaw and Knowles, 1995). Chromosome 11 analysis in this study focused on these regions. The frequency of LOH was compared at 11p13, 11p15, 11q13 and 11q23 in index carcinomas from NR and REC patients. There was more frequent LOH at D11S490 (11q23) in index REC carcinomas than NR carcinomas ($P = 0.004$). Chromosomal deletions at region 11q23, have been associated with human carcinomas including lung (Iizuka et al, 1995), ovarian (Foulkes et al, 1993) and uterine cervix (Hampton et al, 1994), suggesting the presence of at least one tumour suppressor gene in this area. Several candidate genes linked to carcinogenesis at 11q23 include *Cbl*, *EST1*, *DDX6*, tumour suppressor gene on chromosome 11 (*TSG11*) (locus link) and *LOH11CRA* (Monaco et al, 1997). The two most likely candidate genes are *Cbl* and *TSG11*. Cbl protein is a central player in the regulation of tyrosine kinase signalling pathways and is therefore implicated in many growth and apoptotic pathways (Lupher et al, 1998). *TSG11* has been demonstrated as a tumour suppressor gene in lung cancer and possibly a variety of other cancers (Murakami et al, 1998). Although *LOH11CRA* is hypothesized as a possible carcinoma suppressor gene its function is unknown. Further investigations are required but it appears that deletion of 11q23 represents an important genetic event in the recurrence of bladder cancer.

In TCC of the bladder LOH studies on chromosome 17 have tended to focus on 17p13 where *TP53* lies, there is therefore relatively little information available on loss of 17q. Losses at 17q11–22 and 17q24–25 have, however, been reported (Chaturvedi et al, 1997). The current study therefore investigated LOH on chromosome 17 using 18 microsatellites that span the chromosome, 8 of these markers are on 17q. The frequency of LOH at individual markers spanning chromosome 17 ranged from 7% to 80%

© 2001 Cancer Research Campaign depending on the locus investigated. D17S928 (17q25) had the highest frequency of LOH, and was associated with recurrence. Loss of 17q25 is found in breast and ovarian carcinomas (Kalikin et al, 1996, 1997) and is independent of *BRCA 1* deletions. LOH and functional suppression studies in breast, ovarian and oesophageal cancer, support the existence of a novel tumour suppressor gene at 17q24–25 (Jacobs et al, 1993; Godwin et al, 1994; Theile et al, 1995; Plummer et al, 1996; Petty et al, 1998). Candidate genes at 17q24–25 include Thymidine kinase 1 (*TK1*) and apoptosis inhibitor 4 (*API4*). *TK1* encodes soluble thymidine kinase (TK1) which catalyses the phosphorylation of thymidine to deoxythymidine monophosphate and is associated with cell division (Petty et al, 1998). LOH at 17q24–25 is postulated to increase TK1 activity in breast cancer (Petty et al, 1996), and TK1 levels in breast tumours and serum predict recurrence (O'Neill et al, 1992). *AP14* encodes for survivin, which has abundant expression in lung, pancreas, breast and prostate cancer (Ambrosini et al, 1997). Survivin is expressed in G2/M and associates with microtubules of the mitotic spindle at the beginning of mitosis (Li et al, 1998). It is believed to counteract induction of apoptosis in G2/M (Li et al, 1998). Therefore, LOH at this region might interfere with the cell cycle and promote recurrence of TCC of the urinary bladder. Further mapping studies of this area of chromosome 17 and functional studies are required to confirm a role for these candidate genes.

The total frequency of LOH increased with recurrence and progression, demonstrating an accumulation of genetic defects throughout the disease history, but whether this is a cause or a consequence of TCC progression is unknown. Increased LOH with progression was most noticeable at TP53, D17S807, D11S922 and D11S569 (data not shown). Increased LOH at TP53 with carcinoma progression is not surprising as *TP53* alterations are frequently associated with muscle invasivion (Spruck et al, 1994; Sidransky et al, 1991; Chaturvedi et al, 1997).

Genetic differences exist between low grade TCC which may determine subsequent tumour behaviour and identification of the associated tumour suppressor genes is required for better understanding of bladder carcinogenesis.

### ACKNOWLEDGEMENTS

This project was funded by a grant from the Scottish Executive, Health Department Chief Scientific Office.

### REFERENCES

Adshead JM, Kessling AM and Ogden CW (1998) Genetic initiation, progression and prognostic markers in transitional cell carcinoma of the bladder: A summary of the structural and transcriptional changes, and the role of developmental genes. *Br J Urol* **82**: 503–512

Ambrosini G, Adida C and Altieri DC (1997) A novel anti-apoptosis gene, survivin, expressed in cancer and lymphoma. *Nat Med* **3**: 917–921

Bartlett JMS, Watters AD, Ballantyne SA, Going JJ, Grigor KM and Cooke TG (1998) Is chromosome 9 loss a marker of disease recurrence in transitional cell carcinoma of the urinary bladder? *Br J Cancer* **77**: 2193–2198

Bell SM, Zuo J, Myers RM and Knowles MA (1996) Fluorescence in situ hybridisation deletion mapping at 4p16.3 in bladder cancer cell lines refines the localisation of the critical interval to 30 kb. *Genes Chromosom Cancer* **17**: 108–117

Chaturvedi V, Li L, Hodges S, Johnston D, Ro JY, Logothetis C, Von Eschenbach AC, Batsakis JG and Czerniak B (1997) Superimposed histologic and genetic mapping of chromosome 17 alterations in human urinary bladder neoplasia. *Oncogene* **14**: 2059–2070

Czermiak B, Chaturvedi V, Li L, Hodges S, Johnston D, Roy JY, Luthur A, Logothetis C, Von Eschenbach AC, Grossman HB, Benedict WF and Batsakis JG (1999) Superimposed histologic and genetic mapping of chromosome 9 in progression of human urinary bladder neoplasm: implications for a genetic model of multi step urothelial carcinogenesis and early detection of urinary bladder cancer. *Oncogene* **18**: 1185–1196

Edwards J, Duncan P, Going JJ, Watters AD and Bartlett JMS (2000) Loss of hererozgosity on chromosome 9 as a potential marker of recurrence and progression in bladder cancer. *Br J Cancer* **83**(**Suppl 1**): 76 (abstract)

Esrig D, Freeman JA, Stein JP and Skinner DG (1997) Early cystectomy for clinical stage T1 transitional cell carcinoma of the bladder. *Semin Urol Oncol* **15**: 154–160

Fearon ER, Feinberg AP, Hamilton SH and Vogelstein B (1985) Loss of genes on the short arm of chromosome 11 in bladder cancer. *Nature* **318**: 377–380

Foulkes WD, Campbell IG, Stamp GWH and Trowsdale J (1993) Loss of heterozygosity and amplification on chromosome 11q in human ovarian cancer. *Br J Cancer* **67**: 268–273

Godwin AK, Vanderveer L, Schultz DC, Lynch HT, Altomare DA, Buetow KH, Daly M, Getts LA, Mansy A and Rosenblum N (1994) A common region of deletion on chromosome 17q in both sporadic and familial epithelial ovarian tumours distal to BRCA1. *Am J Hum Genet* **55**: 666–677

Going JJ and Lamb RF (1996) Practical histological microdissection for PCR analysis. *J Pathol* **179**: 121–124

Hampton GM, Penny LA, Baergen RN, Larson A, Brewer C, Liao S, Busby-Earle RMC, Williams AWR, Steel CM, Bird CC, Stanbridge EJ and Evans GA (1994) Loss of heterozygosity in cervical carcinoma: Subchromosomal localisation of a putative tumour suppressor gene to chromosome 11q22-q24. *Proc Natl Acad Sci USA* **91**: 6953–6957

Iizuka M, Sugiyama Y, Shiraishi M, Jones C and Sekiya T (1995) Allelic losses in human chromosome 11 in lung cancers. *Genes Chromosom Cancer* **13**: 40–46

Jakob IJ, Smith SA, Wiseman RW, Futreal PA, Harrington T, Osborne RJ, Leech V, Molyneux A, Berchuck A and Ponder BAJ (1993) A deletion unit on chromosome 17q in epithelial ovarian tumours distal to the familial breast/ovarian cancer locus. *Cancer Res* **53**: 1218–1221

Kalikin LM, Qu X, Frank TS, Caduff RF, Svoboda SM, Law DJ and Petty EM (1996) Detailed deletion analysis of sporadic breast carcinomas defines an interstitial region of allelic loss on 17q25. *Genes Chromosom Cancer* **17**: 64–68

Kalikin LM, Frank TS, Svoboda-Newman SM, Wetzel JC, Cooney KA and Petty EM (1997) A region of interstitial 17q25 allelic loss in ovarian carcinomas coincides with a defined region of loss in breast carcinomas. *Oncogene* **14**: 1991–1994

Knowles MA (1999) Identification of novel bladder tumour suppressor genes. *Electrophoresis* **20**: 269–279

Knowles MA (1995) Molecular genetics of bladder cancer: Pathways of development and progression. *Br J Urol* **75**(**Suppl 11**): 57–66

Li F, Ambrosinin G, Chu EY, Plescia J, Tognin S, Marchisio PC and Altieri DC (1998) Control of apoptosis and mitotic spindle checkpoint by survivin. *Nature* **396**: 580–584

Locus Link www.ncbi.nlm.nih.gov/locuslink/list.cgi.

Lupher ML, Rao N, Lill NL, Andoniou CE, Miyake S, Clark EA and Druker BH (1998) Cbl-mediated negative regulation of the Syk tyrosine kinase. A critical role for Cbl phosphotyrosine-binding domain binding to Syk phosphotyrosine 323. *J Biol Chem* **273**: 35273–35281

Monaco C, Negrini M, Sozzi G, Veronese ML, Vorechovsky I, Godwin AK and Croce CM (1997) Molecular cloning and characterisation of LOH11CR2A, a new gene within a refined minimal region of LOH at 11q23. *Genomics* **46**: 217–222

Murakami Y, Nobukuni T, Tamura K, Maruyama T, Sekiya T, Arai Y, Gomyou H, Tanigami A, Ohki M, Cabin D, Frischmeyer P, Hunt P and Reeves RH (1998) Localisation of tumour suppressor activity important in nonsmall cell lung carcinoma on chromosome 11q. *Proc Natl Acad Sci USA* **95**: 8153–8158

Niederacher D, Picard F, van Roeyen C, An H-X, Bender HG and Beckmann MW (1997) Patterns of allelic loss on chromosome 17 in sporadic breast carcinomas detected by fluroescent labelled microsatellite analysis. *Genes Chromosom Cancer* **18**: 181–192

Nishiyama H, Hornigold NH, Davies AM and Knowles MA (1999) A sequence ready 840-kb PAC contig spanning the candidate tumour suppressor locus DBC1 on human chromosome 9q32-q33. *Genomics* **59**: 335–338

Ohgaki K, Iinda A, Ogawa O, Kubota Y, Akimoto M and Emi M (1999) Localisation of tumour suppressor gene associated with distant metastasis of urinary bladder cancer to a 1-Mb interval on 8p22. *Genes Chromosom Cancer* **25**: 1–5

O'Neill KL, McKelvey VJ, Hoper M, Monteverde H, Odlong-Smee GW, Logan H, Abram WP and McKenna PG (1992) Breast tumour thymidine kinase levels and disease recurrence. *Med Lab Sci* **49**: 244–247

*© 2001 Cancer Research Campaign*

Petty EM, Miller DE, Grant AL, Collins EE, Glover TW and Law DJ (1996) FISH localisation of the soluble thymidine kinase gene (TK1) to human 17q25, a region of chromosomal loss in sporadic breast tumours. *Cytogenet Cell Genet* **72**: 319–321

Petty EM, Kalikin LM, Orringer MB and Beer DG (1998) Distal chromosome 17q loss in Barretts esophageal adenocarcinomas and gastric cardia adenocarcinomas: implication for tumourgenesis. *Mol Carc* **22**: 222–228

Plummer SJ, Adams L, Simmons JA and Casey G (1997) Localisation of a growth suppressor activity in MCF7 breast cancer cells to chromosome 17q24–25. *Oncogene* **14**: 2339–2345

Richter J, Jiang F, Gorog JP, Sartonius G, Egerter C, Gasser TC, Moch H, Mihatsch MJ and Sauter G (1997) Marked genetic differences between stage pTa and stage pT1 papillary bladder cancer detected by comparative genomic hybridisation. *Cancer Res* **57**: 2860–2864

Sauter G, Carroll P, Moch H, Kailioniemi A, Kerschmann R, Narayan P, Mihatsch MJ and Waldman FM (1995) c-myc copy number gains in bladder cancer detected by fluorescence in situ hybridisation. *Am J Pathol* **146**: 1131–1139

Sidransky D, Von Eschenbach A, Tsai YC, Jones P, Summerhayes I, Marshall F, Paul M, Green P, Hamilton SR, Frost P and Vogelstein B (1991) Identification of p53 gene mutations in bladder cancers and urine samples *Science* **252**: 706–709

Simon R, Burger H, Brinkschmidt C, Bocker W, Hertle L and Terpe HJ (1998) Chromosomal aberrations associated with invasion in papillary superficial bladder cancer. *J Pathol* **185**: 345–351

Shaw ME and Knowles MA (1995) Deletion mapping of chromosome 11 in carcinoma of the bladder. *Genes Chromosom Cancer* **13**: 1–8

Spruck CH, Ohneseit PF, Gonzalez-Sulueta M, Esrig D, Noriomi M, Tsai YC, Lerner SP, Schmutte A, Yang AS, Cote R, Dubeau LD, Nichols PW, Hermann GG, Steven K, Horn T, Skinner DG and Jones PA (1994) Two molecular pathways to transitional cell carcinoma of the bladder. *Cancer Res* **54**: 784–788

Theile M, Hartman S, Scherthan H, Arnold W, Deppert W, Frege R, Glaab RF, Haensch W and Scherneck S (1995) Suppression of tumorigenicity of breast cancer cells by transfer of chromosome 17 does not require transferred BRCA1 and p53 genes. *Oncogene* **10**: 439–447

Tsutsumi M, Sugano K, Yamaguchi K, Kakizoe T and Akaza H (1997) Correlation of allelic loss of the P53 gene and tumour grade, stage, and malignant progression in bladder cancer. *Int J Urol* **4**: 74–78

Van der Meijden APM (1999) Bladder cancer. *BMJ* **317**: 1366–1369

Voorter CE, Ummelen Mi, Ramaekers FS and Hopman AH (1996) Loss of chromosome 11 and 11p/q imbalances in bladder cancer detected by fluorescence in situ hybridisation. *Int J Cancer* **65**: 301–307

Wagner U, Bubendorf L, Gasser TC, Moch H, Gorog JP, Richter J, Mihatsch MJ, Waldman FM and Sauter G (1997) Chromosome 8p deletions are associated with invasive tumour growth in urinary bladder cancer. *Am J Pathol* **151**: 753–759

Wagner U, Suess K, Luginbuhl T, Schmid U, Ackermann D, Zellweger T, Maurer R, Alund G, Knonagel H, Rist M, Jordan P, Moch H, Mihatsch MJ, Gasser TC and Sauter G (1999) Cyclin D1 overexpression lacks prognostic significance in superficial urinary bladder cancer. *J Pathol* **188**: 44–50

Watters AD, Ballantyne SA, Going JJ, Grigor KM and Bartlett JMS (2000) Aneusomy of chromosomes 7 and 17 predicts the recurrence of transitional cell carcinoma of the urinary bladder. *Br J Urol* **85**: 42–47

*© 2001 Cancer Research Campaign*
